# The Interaction of Munc18-1 Helix 11 and 12 with the Central Region of the VAMP2 SNARE Motif Is Essential for SNARE Templating and Synaptic Transmission

**DOI:** 10.1523/ENEURO.0278-20.2020

**Published:** 2020-11-04

**Authors:** Timon André, Jessica Classen, Philipp Brenner, Matthew J. Betts, Bernhard Dörr, Susanne Kreye, Birte Zuidinga, Marieke Meijer, Robert B. Russell, Matthijs Verhage, Thomas H. Söllner

**Affiliations:** 1 Heidelberg University Biochemistry Center, Heidelberg 69120, Germany; 2 Department of Functional Genomics; 3 Clinical Genetics, Center for Neurogenomics and Cognitive Research (CNCR) Amsterdam Neuroscience, VU University and University Medical Center Amsterdam (UMCA), Amsterdam 1081HV, The Netherlands; 4 BioQuant, Heidelberg University, Heidelberg 69120, Germany

**Keywords:** crosslinking, membrane fusion, Munc18-1, neurotransmission, SNARE, VAMP2

## Abstract

Sec1/Munc18 proteins play a key role in initiating the assembly of N-ethylmaleimide-sensitive factor attachment protein receptor (SNARE) complexes, the molecular fusion machinery. Employing comparative structure modeling, site specific crosslinking by single amino acid substitutions with the photoactivatable unnatural amino acid p-Benzoyl-phenylalanine (Bpa) and reconstituted vesicle docking/fusion assays, we mapped the binding interface between Munc18-1 and the neuronal v-SNARE VAMP2 with single amino acid resolution. Our results show that helices 11 and 12 of domain 3a in Munc18-1 interact with the VAMP2 SNARE motif covering the region from layers −4 to +5. Residue Q301 in helix 11 plays a pivotal role in VAMP2 binding and template complex formation. A VAMP2 binding deficient mutant, Munc18-1 Q301D, does not stimulate lipid mixing in a reconstituted fusion assay. The neuronal SNARE-organizer Munc13-1, which also binds VAMP2, does not bypass the requirement for the Munc18-1·VAMP2 interaction. Importantly, Munc18-1 Q301D expression in *Munc18-1* deficient neurons severely reduces synaptic transmission, demonstrating the physiological significance of the Munc18-1·VAMP2 interaction.

## Significance Statement

Reliable neurotransmitter release depends on the precisely controlled Ca^2+^-synchronized fusion of synaptic vesicles with the presynaptic plasma membrane. This requires the correct assembly of the molecular membrane fusion machinery, N-ethylmaleimide-sensitive factor attachment protein receptor (SNARE) complexes, by Sec1/Munc18 proteins. In this study, we establish a map with single amino acid precision of how Munc18-1 provides a template for the binding of the v-SNARE protein VAMP2 and demonstrate its relevance for synaptic function.

## Introduction

Sec1/Munc18 (SM) family members control soluble N-ethylmaleimide-sensitive factor attachment protein receptor (SNARE) complex assembly at most vesicular transport steps (Toonen and Verhage, 2003; Südhof and Rothman, 2009; Carr and Rizo, 2010; Rizo and Südhof, 2012). V-SNAREs and t-SNAREs on opposing membranes (vesicular and target membrane, respectively) form cognate trans-SNARE complexes and their assembly into a four-helix bundle provides the energy to drive membrane fusion (Sutton et al., 1998; Weber et al., 1998; Sørensen et al., 2006; Gao et al., 2012; Min et al., 2013). Four-helix bundle formation is mediated by SNARE motifs that consist of 15–16 hydrophobic layers characterized by a central “0 layer” usually containing an arginine (R) in the v-SNARE and glutamines (Qa, Qb, Qc) in the t-SNAREs (Sutton et al., 1998).

**Figure 1.**
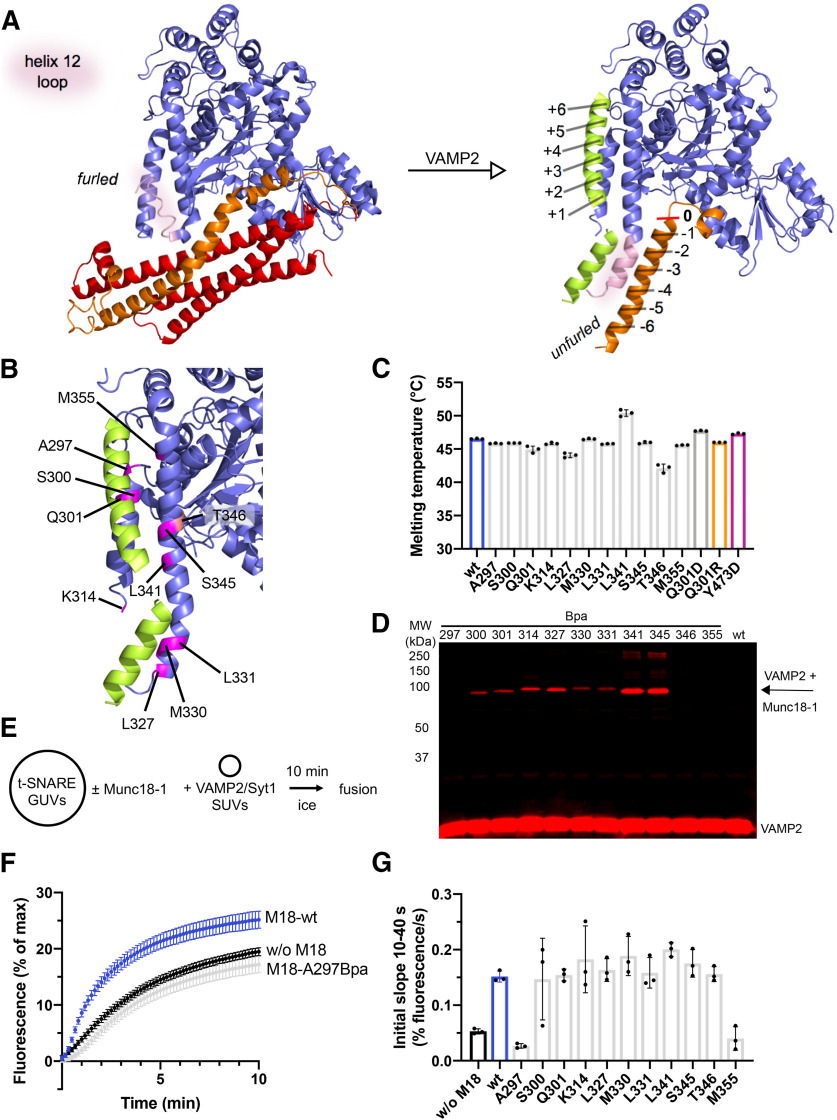
Layers −4 to +4 of VAMP2 bind to helix 11 and helix 12 of Munc18-1. ***A***, Crystal structure (PDB:3C98) of Munc18-1 (blue) bound to the inhibitory, closed conformation of syntaxin-1 (Habc-domain: red, SNARE motif: orange). The loop connecting helix 11 and 12 of Munc18-1 (pink) is in a furled conformation. Structural model of the template complex based on the *Chaetomium thermophilum* Vps16·Vps33·Nyv1 (PDB:5BV0) and the Vps16·Vps33·Vam3 (PDB: 5BUZ) structures. Layers (−6 to +6) in the SNARE motif are assigned according to Fasshauer et al. (1998). ***B***, Predicted Munc18-1·VAMP2 interface. Munc18-1 amino acids that should contribute to VAMP2 binding are shown in purple. T346 (salmon) should not interact with VAMP2. ***C***, Munc18-1 wt and Munc18-1 point mutants show similar melting temperatures. ***D***, Site-specific Munc18-1·VAMP2 crosslink products were analyzed by Western blotting using an anti-VAMP antibody. ***E***, Incubation scheme of t-SNARE GUV based liposome fusion assay. ***F***, Representative kinetic traces of the lipid mixing assay. VAMP2/Syt1 SUVs labeled with Atto488-DPPE/Atto550-DPPE were incubated with syntaxin-1/SNAP-25 GUVs labeled with Atto647-DPPE in the absence or presence of the indicated Munc18-1 (M18) constructs. Lipid mixing was monitored at 37°C for 10 min by an increase of Atto488 fluorescence. ***G***, Quantification of all tested Munc18-1 Bpa mutants except A297Bpa and M355Bpa shows lipid mixing stimulation comparable to Munc18-1 wt. Bar graph represents the initial slope from 10 to 40 s (% fluorescence/s). Error bars: SD (*n* = 3) of technical replicates.

SNARE complex assembly is controlled by a cascade of reactions involving multiple proteins, arresting or accelerating the formation of distinct intermediates and promoting unidirectionality (for review, see Rizo, 2018; Brunger et al., 2019). SM proteins increase the specificity of SNARE interactions and accelerate SNARE complex formation (Shen et al., 2007). A decisive and rate-limiting step is the formation of a SM*·*Qa-SNARE*·*R-SNARE complex that provides the template for the binding of the other two SNARE motifs (Baker et al., 2015). To date, detailed structural information about such critical intermediates is limited to components of the endosomal/vacuolar machinery. Two crystal structures show the SM protein Vps33 with either the Qa-SNARE Vam3 or with the R-SNARE Nyv1 (Baker et al., 2015). Structural information about other SM proteins is restricted to neuronal Munc18 in the absence or the presence of syntaxin (Qa-SNARE; Misura et al., 2000; Bracher et al., 2002; Hu et al., 2007). The Munc18-1*·*syntaxin-1 crystal structure represents the inhibitory mode (Misura et al., 2000; Burkhardt et al., 2008). Here, the regulatory N-terminal Habc domain of syntaxin-1 folds back onto its SNARE motif and is stabilized by Munc18-1 (Dulubova et al., 1999; Misura et al., 2000; Fig. 1). This closed Munc18-1·syntaxin-1 complex needs structural rearrangements for the R-SNARE VAMP2 and the Qb,c-SNARE SNAP-25 to be incorporated into the SNARE complex. The other Munc18 crystal structure likely resembles the active conformation but lacks syntaxin-1 and VAMP2 (Hu et al., 2007). In the inhibitory conformation, a furled loop connecting helices 11 and 12 in domain 3a of Munc18-1 is folded back on domain 3a. In the activating conformation this loop is unfurled and extends helix 12 (Fig. 1) which is sterically incompatible with binding of closed syntaxin-1. VAMP2 promotes the active conformation (Shen et al., 2007; Schollmeier et al., 2011; Munch et al., 2016; Meijer et al., 2018) by inducing helix 12 extension and formation of a “template complex” with an open syntaxin-1 conformation (Jiao et al., 2018). Distinct mutations in domain 3a that favor the open conformation of Munc18-1 yield gain of function phenotypes (Parisotto et al., 2014; Sitarska et al., 2017; Jiao et al., 2018). Importantly, Munc13-1 assists template complex formation by inducing a conformational change within the syntaxin-1 linker region between the Habc and SNARE domains (Ma et al., 2011, 2013; Yang et al., 2015; Wang et al., 2017; Magdziarek et al., 2020). Furthermore, Munc13-1 stabilizes the template complex by interacting with the membrane proximal linker region of VAMP2 (Wang et al., 2019; Shu et al., 2020).

To provide mechanistic insights into Munc18-1 function in the natural membrane environment, we modeled the Munc18-1*·*VAMP2 structure and introduced a photoactivatable unnatural amino acid at defined positions in Munc18-1 to map the interaction site for membrane anchored VAMP2. Based on our analysis, we generated a new loss of function (Q301D) and a new gain of function (Q301R) mutant in helix 11. The mutants significantly influenced VAMP2 binding in opposite directions and caused pronounced phenotypes in *in vitro* reconstituted membrane fusion, which persisted in the presence of Munc13-1. Consistently, the Q301D mutant led to a loss of function phenotype in synaptic transmission, when expressed in Munc18-1 deficient neurons. Together, these data define the Munc18-1*·*VAMP2 interface and demonstrate its role in regulated exocytosis.

## Materials and Methods

### Munc18-1 mutagenesis

DNA constructs were made using standard genetic manipulations. Munc18-1 cDNA subcloned into the pEG(KG) vector (a kind gift of Dr. Richard Scheller) was mutated by QuikChange site-directed mutagenesis (Stratagene) using Phusion polymerase (New England Biolabs) and the following HPLC-purified mutagenesis primers:

A297tag forward (5′-ctgcgacacaagcacatctaggaggtgtcccaggaagtg-3′), A297tag reverse (5′-cacttcctgggacacctcctagatgtgcttgtgtcgcag-3′), S300tag forward (5′-cacatcgcagaggtgtagcaggaagtgacccgg-3′), S300tag reverse (5′-ccgggtcacttcctgctacacctctgcgatgtg-3′), Q301tag forward (5′-cgggtcacttcctaggacacc tctgcg-3′), Q301tag reverse (5′-cgcagaggtgtcctaggaagtgacccg-3′), K314tag forward (5′-ctccagtattcatcctctagctagaggaaaagtcctt-3′), K314tag reverse (5′-aaggacttttcctctagctagaggatgaatactggag-3′), L327tag forward (5′-gcattttcttcagcatctgggactagtcccgcatggtggtcttc-3′), L327tag reverse (5′- gaagaccaccatgcgggactagtcccagatgctgaagaaaatgc-3′), M330tag forward (5′-catgcgggacctgtcccagtagctgaagaaaatgccccag-3′), M330tag reverse (5′-ctggggcattttcttcagctactgggacaggtcccgcatg-3′), L331tag forward (5′-actggggcattttcttctacatctgggacaggtccc-3′), L331tag reverse (5′-gggacctgtcccagatgtagaagaaaatgccccagt-3′), L341tag forward (5′-gccccagtaccagaaggagtagagcaagtattcgactcacc-3′), L341tag reverse (5′-ggtgagtcgaatacttgctctactccttctggtactggggc-3′), S345tag forward (5′-cagaaggagctcagcaagtattagactcacctgcaccttg-3′), S345tag reverse (5′-caaggtgcaggtgagtctaatacttgctgagctccttctg-3′), T346tag forward (5′-ggagctcagcaagtattcgtagcacctgcaccttgctgaag-3′), T346tag reverse (5′-cttcagcaaggtgcaggtgctacgaatacttgctgagctcc-3′), M355tag forward (5′-caccttgctgaagactgttagaagcattaccaaggcacc-3′), M355tag reverse (5′-ggtgccttggtaatgcttctaacagtcttcagcaaggtg-3′), Q301R forward (5′-gcacatcgcagaggtgtcccgggaagtgacccggt-3′), Q301R reverse (5′-gaccgggtcacttcccgggacacctctgcgatgtgc-3′), Q301D forward (5′-gaccgggtcacttcatcggacacctctgcga-3′), and Q301D reverse (5′-tcgcagaggtgtccgatgaagtgacccggtc-3′).

The integrity of the mutants was verified by DNA sequencing at LGC Genomics.

### Protein purification

Mammalian glutathione-S-transferase (GST)-tagged or His6-tagged proteins were expressed in *Escherichia coli* BL21 (DE3; Stratagene). Protein purification of GST-VAMP2, GST-VAMP8, His6-syntaxin-1, His6-SNAP-25, t-SNARE (syntaxin-1·His6-SNAP-25), His6-Synaptotagmin-1 (Syt1), and GST-Munc18-1 wt was performed as described previously via glutathione Sepharose 4 fast flow (GE Healthcare Biosciences) or Ni^2+^-NTA (QIAGEN) affinity chromatography and in some cases subsequent ion exchange chromatography (Weber et al., 1998; Malsam et al., 2012; Parisotto et al., 2014; Schollmeier et al., 2011; Meijer et al., 2018). The GST-Munc18-1 wt, Q301D/R, and Y473D point mutants were expressed and purified as described previously (Parisotto et al., 2014).

The GST-Munc18-1 p-Benzoyl-phenlyalanine (Bpa) point mutants were expressed using the pEVOL/pET expression system (Young et al., 2010). To this end, *E. coli* BL21 were co-transformed with the pEVOL-pBpF plasmid and the expression plasmid carrying the respective Munc18-1 amber mutant. Cells were grown in 1 l LB media supplemented with ampicillin and chloramphenicol over night at 20°C. The next day, the incubation temperature was set to 37°C. At an OD_600 nm_ of 0.6, a final concentration of 1 mm Bpa was added (from 100× Bpa stock dissolved in 1 ml 1 M NaOH). The suspension was cooled to 30°C and the cells were grown for 1 h. Then arabinose (0.25% final concentration) was added and the cells were grown at 30°C for 1 h. Expression of Munc18-1 Bpa mutants was induced by adding a final concentration of 0.3 mm isopropyI-β-D-thiogalactopyranoside (IPTG) at 22°C overnight. Purification proceeded as described previously (Parisotto et al., 2014).

In order to express rat Munc13-1-C1C2BMUN, a DNA sequence encoding Munc13-1 with the same loop substitution as described previously (Ma et al., 2013; amino acids 526-1407_EF_1453-1531) was generated and cloned into a pPROEX1 plasmid with a TEV cleavable N-terminal His6 tag. Protein expression in BL21DE3 *E. coli* cells was induced with 1% w/v lactose in LB medium and conducted over night at 16°C. Cells were harvested by centrifugation at 3000 rcf for 15 min, washed with ice cold PBS and resuspended in lysis buffer [25 mm HEPES-KOH (pH 7.4), 400 mm KCl, 10% glycerol, 1% Triton X-100, 25 mm imidazole, and 1 mm dithiothreitol (DTT)]. Final concentrations of 1 mm MgCl_2_, 25 U/ml benzonase, and an EDTA-free protease inhibitor mix (25 μg/ml turkey trypsin inhibitor, 12.5 μg/ml benzamidine, 6.25 μg/ml Pefabloc SC, 2.5 μg/ml antipain, 1.5 μg/ml leupeptin, 1.25 μg/ml aprotinin, 5 μg/ml chymostatin, and 2.5 μg/ml pepstatin A) were added to the cell suspension while stirring and incubated for 10 min on ice. Cells were lysed with a M-110L homogenizer (Microfluidics) in one passage at 18,000 psi and the lysate was cleared by centrifugation at 100,000 rcf for 30 min. Ni-NTA agarose equilibrated with lysis buffer was incubated with cleared lysate for 1–2 h at 4°C. Loaded beads were washed with buffer A [25 mm HEPES-KOH (pH 7.4), 135 mm KCl, 10% glycerol, 50 mm imidazole, and 1 mm DTT] followed by buffer A containing 70 mm imidazole. The protein was eluted in buffer B [25 mm HEPES-KOH (pH 7.4), 135 mm KCl, 10% glycerol, 200 mm imidazole, and 1 mm DTT]. The eluate was dialyzed against fusion buffer [25 mm HEPES-KOH (pH 7.4), 135 mm KCl, and 1 mm DTT] over night at 4°C, insoluble material was removed by centrifugation at 20,000 rcf for 10 min, and the supernatant was snap frozen in liquid nitrogen.

### Nano-differential scanning fluorimetry (nanoDSF)

To assess protein stability, the melting curves of Munc18-1 wt and of the point mutants were recorded using the Prometheus NT.48 instrument (NanoTemper Technologies). Standard capillaries (PR-C002) were filled with ∼10-μl sample. A temperature gradient of 1°C min^−1^ or 1.5°C min^−1^ from 25°C to 95°C was applied, and the intrinsic protein fluorescence at 330 and 350 nm was recorded. The melting points were calculated using the default configurations of the nanoDSF software (PR. ThermControl, version 2.1.2).

### Protein reconstitution into liposomes

All lipids used were purchased from Avanti Polar lipids. Proteins were reconstituted into small unilamellar vesicles (SUVs) as described previously (Malsam et al., 2012; Parisotto et al., 2014).

For the v-SNARE/Syt1 SUVs, the following lipid composition was used: 29 mol% 1-palmitoyl-2-oleoyl-SN-glycero-3-phosphocholine (POPC), 15 mol% 1,2-dioleoyl-SN-glycero-3-phosphoserine (DOPS), 25 mol% 1-hexadecanoyl-2-octadecenoyl-SN-glycero-3-phosphoethanolamine (POPE), 5 mol% liver L-α-phosphatidylinositol (PI), 25 mol% cholesterol (from ovine wool), 0.5 mol% Atto488-1,2-dipalmitoyl-sn-glycero-3-phosphoethanolamine (DPPE), 0.5 mol% Atto550-DPPE. For each reconstitution 3 μmol total lipid was used. VAMP2 or VAMP8 were incorporated with a protein-to-lipid ratio of 1:300 and Syt1 1:800.

The syntaxin-1 or t-SNARE SUVs for subsequent giant unilamellar vesicle (GUV) formation, consisted of 34.95 mol% POPC, 15 mol% DOPS, 20 mol% POPE, 3 mol% liver PI, 2 mol% brain PI(4,5)P2 (L-α-phosphatidylinositol-4,5-bisphosphate), 25 mol% cholesterol, and 0.05 mol% Atto647-DPPE. For each reconstitution, 5 μmol total lipid was used. Syntaxin-1 or t-SNARE (syntaxin-1/SNAP-25) was reconstituted with a protein-to-lipid ratio of 1:1000. Subsequently, GUVs were formed by electro-swelling as described previously (Malsam et al., 2012) with the following modifications: (1) the second desalting step was done using a PD10 column (GE Healthcare); (2) instead of ITO-coated glass slides platinum-coated slides were used; and (3) the centrifugation step after detaching the GUVs from the glass slides was omitted.

Protein-to-lipid ratios of the reconstituted liposomes were determined by correlating the lipid amounts, quantified via Atto488 or Atto647 fluorescence, and the protein amounts. Protein amounts were quantified by SDS–PAGE and staining with Coomassie Blue, using a bovine serum albumin (BSA) protein standard. The ImageJ software was used to determine the gray values of the respective protein bands.

### Site-specific photo-crosslinking

A total 1 μm Munc18 wt or a respective Munc18-1 Bpa mutant was incubated with syntaxin-1 GUVs (28 nmol lipid) in a total volume of 90 μl for 30 min at room temperature (RT). Subsequently, 10-μl VAMP2/Syt1 SUVs (5 nmol lipid) were spun at 20,000 × *g* for 5 min to remove larger SUV complexes and the supernatant was added to the GUV suspension. Following an incubation on ice for 30 min, the liposome suspension was layered on top of a 100-μl “sucrose cushion” containing a 4:1 mixture of fusion buffer [25 mm HEPES (pH 7.4), 135 mm KCl, and 1 mm DTT] and sucrose buffer (1 mm HEPES, 240 mm sucrose, and 1 mm DTT) and spun at 10,000 rpm for 10 min in a SW55Ti rotor (Beckman). A total of 190 μl of the supernatant was discarded, and the remaining volume was resuspended and transferred into a metal block. The samples were illuminated on ice with a UV LED smart SN1212 lamp (Opsytec) for 15 pulses × 1 s with 2-s pauses. Then, the samples were analyzed by SDS-PAGE and Western blotting and probed for crosslink products by immunodecoration with antibodies (anti VAMP2 1:1000, Parisotto et al., 2014; Malsam et al., 2020).

### SUV/GUV fusion assay

The lipid-mixing assay using GUVs that contain the preassembled t-SNARE complex was performed as described previously (Malsam et al., 2012; Parisotto et al., 2014; Meijer et al., 2018) with the following modifications: (1) 500 nm Munc18-1 wt or the respective mutants were used, (2) CaCl_2_ addition was omitted. The fluorescence signal of each measurement was normalized to the respective maximal signal obtained after lysis of the liposomes by addition of SDS and dodecylmaltoside (0.8% final concentration each). Further, the fluorescence signals of control reactions containing GUVs preincubated with the soluble part of VAMP2 1–94 (VAMP2 CD) were subtracted from each measurement.

The lipid-mixing assay using GUVs that only contain syntaxin-1 was performed as described previously (Munch et al., 2016). Briefly, syntaxin-1 GUVs (14 nmol lipid) were preincubated in the absence or presence of 500 nm Munc18-1 wt, Munc18-1 Q301D, or Munc18-1 Q301R for 30 min at RT. Subsequently, SNAP-25 (1.7 μm) and VAMP2/Syt1 SUVs (2.5 nmol lipid) were added on ice in a total volume of 100 μl for 10 min to allow vesicle docking. Samples were transferred into a 96-well microtiter plate, the temperature was raised to 37°C and membrane fusion was measure by the increase of Atto488 fluorescence in a fluorescence plate reader (BioTek). The signal of each incubation was normalized to the respective maximal signal obtained after lysis of the liposomes by SDS and dodecylmaltoside (0.8% final concentrations). Fluorescence traces of negative-control reactions containing GUVs treated with botulinum neurotoxin C (BoNTC) were subtracted from each measurement.

For lipid-mixing experiments in presence of 500 nm C1C2BMUN, syntaxin-1 GUVs containing 33.9 mol% POPC, 15 mol% DOPS, 20 mol% POPE, 4 mol% liver PI, 1 mol% brain PI(4,5)P2, 25 mol% cholesterol, 1 mol% diacylglycerol (DAG) and 0.1 mol% Atto647-DPPE were used. Syntaxin-1 GUVs (14 nmol lipid) were preincubated in the absence or presence of Munc18-1 wt, Munc18-1 Q301D, or Munc18-1 Q301R for 30 min at RT. Afterwards, C1C2BMUN-wt or mutants were added and incubated for 5 min on ice. Subsequently, SNAP-25 and VAMP2/Syt1 SUVs (2.5 nmol lipid) were added in a final volume of 100 μl for 15 min to allow vesicle docking. Samples were transferred into a 96-well microtiter plate at 37°C and membrane fusion was measured by the increased Atto488 fluorescence in a fluorescence plate reader. The signal of each incubation was normalized to the respective maximal signal obtained after lysis of the liposomes by SDS and dodecylmaltoside (∼0.8% final concentrations). Fluorescence traces of negative-control reactions lacking SNAP-25 were subtracted from each measurement. Fluorescence traces were further normalized to the fluorescence at time x = 0 min. Please note that for measurements containing the Munc13-1 constructs, the syntaxin-1 GUVs (used in Fig. 4) contain a different lipid composition, in particular 1% DAG, which can lead to some variance in the fusion efficiency. Moreover, compared with Figure 3*B*, the fusion signals shown in Figure 4*C* are reduced. This is likely caused by the (early) addition of Ca^2+^. During the initial incubation only a fraction of VAMP2/Syt1 SUVs have docked and only a fraction of Syt1 molecules has formed functional contacts with PIP2/SNAREs on the GUV surface (Fig. 4*C*). If Ca^2+^ is added to the fusion reaction, Syt1 in the non-associated VAMP2/Syt1-SUVs will bind in *cis* to the SUV membrane, thus overall reducing the functional pool of available SUVs (Stein et al., 2007). This will reduce fusion signals at later time points.

### SUV/GUV co-sedimentation assay

Co-sedimentations were performed as described by (Parisotto et al., 2012), but SNAP-25 was omitted from the incubations. Briefly, before compiling the samples, potential SUV aggregates were removed by centrifugation at 20,000 × *g* for 5 min. Syntaxin-1 GUVs (28 nmol lipid) were preincubated for 10 min at RT in the absence or presence of 500 nm Munc18-1 wt or the indicated point mutants in 180 μl buffer [25 mm HEPES-KOH (pH 7.4), 135 mm KCl, 0.1 mm EGTA, 1 mm MgCl_2_, and 1 mm DTT]. Subsequently, 20 μl of the precleared VAMP2 SUVs (5 nmol lipid) were added to GUVs in a final volume of 200-μl buffer and incubated on ice for 10 min. In order to isolate the docked SUVs, GUVs were isolated by centrifugation for 10 min at 1000 × *g* followed by 10 min at 3000 × *g*; 190 μl of the supernatant was discarded, and the remaining sample was resuspended in a 96-well plate with a final concentration of 0.8% SDS and dodecylmaltoside to determine the fluorescence signals. The background fluorescence of samples containing only VAMP2 SUVs (usually 5% of the input) was subtracted from all samples, and the percentage of co-sedimented SUVs was calculated based on input measurements. The recovery of sedimented GUVs was also calculated based on input measurements. Average values of three independent experiments were calculated using GraphPad Prism.

### Neuronal cultures

Animal experiments were approved by the animal ethical committee of the VU University/VU University Medical Centre (license number: FGA 11–03) and are in accordance with Dutch governmental guidelines and regulations. Animals were housed and bred according to institutional, Dutch, and United States governmental guidelines. Munc18-1 deficient mice were generated as described previously (de Vries et al., 2000). *Munc18 null* mutant mice are stillborn and can be easily distinguished from wild-type or heterozygous littermates. Embryonic day (E)18 embryos were obtained by cesarean section of pregnant females from timed mating of heterozygous mice.

### Constructs

Single amino acid substitutions on Q301 in Munc18-1 were generated using Quikchange and verified by sequencing. The Munc18-1 constructs were connected with a T2 linker to Cre-EGFP and cloned into pLenti vectors, ensuring that expression levels of Cre-EGFP and Munc18-1 were similar. Viral particles were produced as previously described (Naldini et al., 1996).

### Dissociated neuronal cultures

Hippocampi from *Munc18-1 null* mice were collected in ice-cold HBSS (Sigma) buffered with 7 mm HEPES (Invitrogen). After removal of the meninges, hippocampi were incubated in Hanks’-HEPES containing 0.25% trypsin (from 10× stock, Invitrogen) for 20 min at 37°C. After washing, neurons were triturated in DMEM, supplemented with 10% fetal calf serum (FCS), 1% nonessential amino acids (NAAs) using a fire-polished Pasteur pipette and counted in a Fuchs–Rosenthal chamber. Neurons were plated in prewarmed Neurobasal medium supplemented with 2% B-27, 1.8% HEPES, 0.25% glutamax, and 0.1% penicillin/streptomycin (all Invitrogen) and infected with lentiviral particles encoding Munc18-1 variants on the same day.

Hippocampal *Munc18-1 null* neurons were plated on micro-islands of rat glia at a density of 15,000 per well in a 12-well plate to achieve autaptic cultures. To generate micro-islands, glass coverslips (Menzel) were etched in 1 m HCl for at least 2 h and neutralized with 1 m NaOH for maximum 1 h, washed thoroughly with MiliQ water, and washed once with 70% ethanol. Coverslips were stored in 96% ethanol and coated with agarose Type II-A (0.0015% in H_2_O, Sigma). Coating was done by spreading a thin layer of agarose solution (heated in a microwave and kept at 55°C during use) with a cotton swab over the entire coverslip. Microdots were created using a custom-made rubber stamp (dot diameter 250 μm) to apply a solution consisting of 0.1 mg/ml poly-D-lysine (Sigma), 0.7 mg/ml rat tail collagen (BD Biosciences), and 10 mm acetic acid (Sigma) by stamping from a wet filter paper (3-mm cellulose chromatography paper, Whatman). Coverslips were UV-sterilized for 20 min before further use. Astrocytes were plated at 6000–8000/well in prewarmed DMEM (Invitrogen) supplemented with 10% FCS, 1% NAAs, and 1% penicillin/streptomycin (all Invitrogen).

### Electrophysiological recording

Autaptic cultures of *Munc18-1 null* neurons were grown for 14–18 d before electrophysiological measurements were performed. Whole-cell voltage-clamp recordings (V_m_ = −70 mV) were performed at RT. Borosilicate glass pipettes (2.5–5 mΩ) filled with 125 mm K^+^-gluconic acid, 10 mm NaCl, 4.6 mm MgCl, 4 mm K2-ATP, 15 mm creatine phosphate, 10 U/ml phosphocreatine kinase, and 1 mm EGTA (pH 7.3, 300 mOsmol). External solution was made up of 10 mm HEPES, 10 mm glucose, 140 mm NaCl, 2.4 mm KCl, 4 mm MgCl_2_, and 2 mm CaCl_2_ (pH 7.3, 300 mOsmol). Only excitatory neurons, identified by decay of the postsynaptic currents, were included. Recordings were acquired with an Axopatch 200B amplifier, Digidata 1440A, and ClampX 10.2 software (Molecular Devices). After whole-cell mode was established, only cells with an access resistance of <15 mΩ and leak current of <500 pA were included in the analysis. Evoked EPSCs were elicited by a 1-ms-long depolarization to 30 mV. Readily releasable pool (RRP) size was estimated using back-extrapolation by making a linear fit through the last 20 pulses of a 40-Hz stimulation train, where intracellular calcium levels are high and synchronous release has been mostly abolished. At this point newly-recruited fusion-capable vesicles are released immediately. This is considered to be a steady-state situation, at which a linear back-extrapolation procedure can be used to estimate the size of the initial RRP (*y-axis* intercept) corrected for vesicle recruitment. Offline analysis was performed using custom-written software routines in MATLAB R2017b (MathWorks).

### Immunocytochemistry

Cover slips from the same 12-well plates used for electrophysiology were used to control Munc18-1 levels as well as assessment of morphology. Cultures were fixed with 3.7% formaldehyde (Electron Microscopy Sciences). After washing with PBS, cells were permeated with 0.5% Triton X-100 for 5 min and incubated in 2% normal goat serum and 0.1% Triton X-100 for 20 min to block non-specific binding. Cells were incubated for 2 h at RT with a primary antibody mixture of polyclonal guinea pig anti-Synaptophysin-1 (1:1000, SYSY), polyclonal chicken anti-MAP2 (1:10,000, Abcam), and polyclonal rabbit anti-Munc18-1 antibodies (1:1000, described previously; de Vries et al., 2000). After washing, cells were incubated for 2 h at RT with secondary antibodies conjugated to Alexa dyes (1:1000, Invitrogen) and washed again. Coverslips were mounted with DABCO-Mowiol (Invitrogen).

### Microscopy

Images of single cells were acquired with a confocal microscope (LSM 510, Carl Zeiss) using a 40× oil immersion objective (NA = 1.3) with 0.7× zoom at 1024 × 1024 pixels and averaged over four scans. Neuronal morphology and protein levels were analyzed using automated image analysis routine (Schmitz et al., 2011).

### 3D structure model of rat VAMP2·Munc18-1·syntaxin-1A complex

We modelled the rat VAMP2*·*Munc18*·*syntaxin-1A complex 3D structure by superposing structures of rat VAMP2 SNARE domain (PDB:1kil chain A; Chen et al., 2002), the open conformation of Munc18-1 (PDB 3puj chain A; Hu et al., 2011), and a comparative model of rat syntaxin-1A on a *Chaetomium* Nyv1 (SNARE domain), Munc18-like, SNAP-receptor like (SNARE domain) complex structural template we made by merging the binary Nyc1*·*Munc18 and Munc18*·*SNAP receptor structures (PDB 5bv0 and PDB 5buz, respectively; Baker et al., 2015) via a superposition of their shared Munc18-like structure. We made the comparative model of rat syntaxin-1A with Modeller (Webb and Sali, 2016) after aligning the rat syntaxin-1A sequence (UniProt P32851/203–238) to the sequence of the *Chaetomium* SNAP-receptor like structure (PDB 5buz chain C; Baker et al., 2015). All structural superpositions were made using the STAMP package (Russell and Barton, 1992).

### Experimental design and statistical analysis

For the reconstituted *in vitro* assay, we used an ordinary one-way ANOVA with Tukey’s test or with Dunnett’s test. The *in vivo* experiments were statistically tested using the Kruskal–Wallis test and Dunn’s multiple comparison test.

## Results

### Site-specific photo-crosslinking maps the interface between VAMP2 and Munc18-1 in the reconstituted template complex

We developed a structural model of the ternary complex consisting of Munc18-1, syntaxin-1, and VAMP2 (the template complex), based on the endosomal/vacuolar complex (for details, see Materials and Methods). Starting points were the two crystal structures of the Vps33*·*Nyv1 complex (PDB: 5BV0) and the Vps33*·*Vam3 complex (PDB: 5BUZ; Baker et al., 2015). To validate the structure-homology model (Fig. 1*A*, right) and to capture potentially transient protein-protein interactions, we employed a crosslink approach introducing the unnatural photoactivatable amino acid Bpa into Munc18-1 at positions targeting the predicted VAMP2-interface (Fig. 1*B*). Importantly, the incorporation of Bpa had only minor effects on protein stability (Fig. 1*C*). L341BpA and T345Bpa slightly increased and decreased the melting temperatures, respectively. Bpa can be photoactivated and primarily reacts with C-H bonds within a radius of ∼3 Å (Dormán and Prestwich, 1994). The molecular interactions were studied in trans ternary Munc18-1·syntaxin-1·VAMP2 complexes bridging two lipid bilayers, thus including the physiological membrane constraints acting on the complex. Therefore, GUVs containing syntaxin-1 were incubated with Munc18-1 wt or Bpa mutants to generate inhibitory Munc18-1·syntaxin-1 complexes. Subsequently, SUVs containing VAMP2 and the Ca^2+^ sensor Syt1 were added. We have previously shown that Syt1 is essential for initial vesicle docking in intact cells (de Wit et al., 2009) and binds PI(4,5)P2 on the GUV surface in a Ca^2+^-independent manner ensuring efficient SUV docking (Malsam et al., 2012; Parisotto et al., 2012). The absence of SNAP-25 prevents further SNARE complex assembly and precludes membrane merger. Western blot analysis of UV-irradiated samples identified bands that correspond to Munc18-1·VAMP2 crosslink products (Fig. 1*D*). As expected, crosslink products were absent in the sample containing Munc18-1 wt. In addition, we were able to resolve the interaction sites at the single amino acid level. For example, shifting the Bpa incorporation from position 345–346 abolished the crosslink to VAMP2, as predicted by the structure homology model (Fig. 1*A*,*B*). Interestingly, all other Bpa mutants, amino acids S300, Q301, K314, L327, M330, L331, L341, S345, except A297 and M355, yielded crosslink products (Fig. 1*D*). The bulky Bpa residue at A297 and M355 may perturb the interaction with VAMP2. To address this hypothesis, we tested each of the Bpa mutants in a well-established reconstituted lipid-mixing assay using GUVs that contained preassembled t-SNARE (synatxin-1/SNAP-25) complexes (Parisotto et al., 2014). Like Munc18-1 wt, almost all Bpa mutants stimulated lipid mixing by a factor of 3–4 (Fig. 1*E–G*). However, A297Bpa and M355Bpa did not show any stimulatory effect indicating that these mutations indeed abolish the Munc18-1·VAMP2 interaction, consistent with the lack of VAMP2 crosslinks (Fig. 1*D–F*).

In summary, these results reveal that in the reconstituted template complex, helix 11 and helix 12 of Munc18-1 together provide the binding surface for VAMP2. The model and the direct crosslinks indicate that the interface encompasses a region at least covering layers −4 to +4 of the VAMP2 SNARE motif (Fig. 1*A*).

### Q301 in helix 11 of Munc18-1 critically contributes to VAMP2 binding

In the next set of experiments, we focused on glutamine 301 in Munc18-1 introducing natural amino acid substitutions instead of Bpa. According to the molecular model (Fig. 1) Q301 is close to D68 in VAMP2 and could contribute electrostatic interactions. Therefore, we predicted that Munc18-1 Q301R reinforces VAMP2 binding by electrostatic attraction and Q301D may weaken the interaction by electrostatic repulsion.

**Figure 2.**
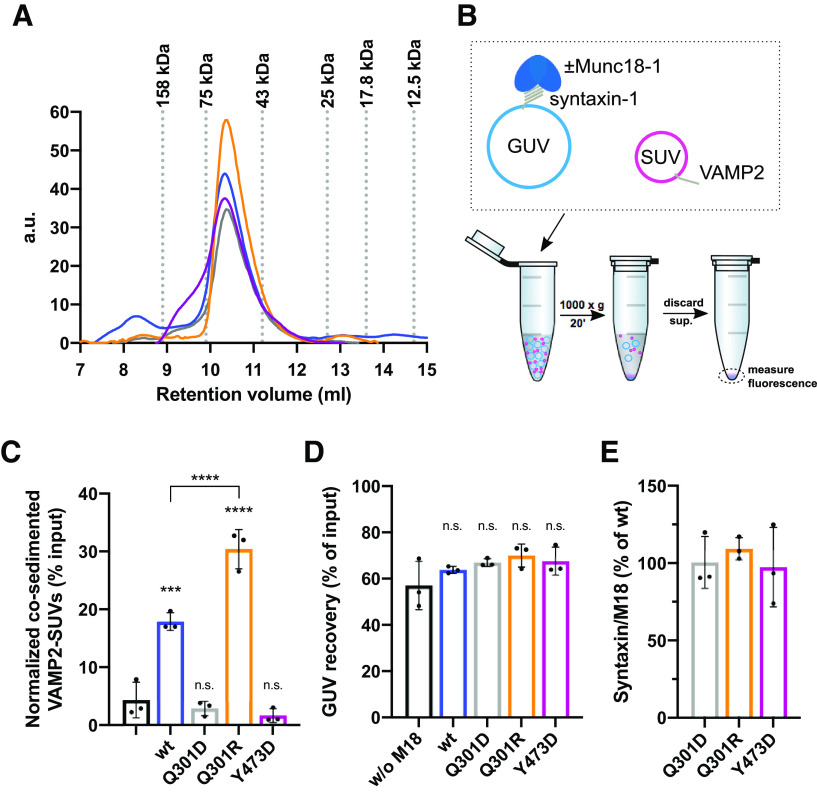
Munc18-1·VAMP2 binding depends on Q301 in helix 11 of Munc18-1. ***A***, Munc18-1 wt and point mutants show the same retention volume in gel filtration experiments. The samples were analyzed on a Superdex 75 Increase 10/300 column (GE Healthcare). ***B***, Schematic representation of the SUV/GUV co-sedimentation assay. ***C***, Munc18-1 Q301R and Q301D show increased and decreased VAMP2 binding, respectively. VAMP2 SUVs labeled with Atto488-DPPE/Atto550-DPPE were incubated with syntaxin-1 GUVs labeled with Atto647-DPPE in the absence or presence of Munc18-1 wt or the indicated single amino acid substitutions. The liposomes were isolated, and their fluorescence was quantified. The normalized amount of co-sedimented SUVs is plotted as % of input. ***D***, GUV recovery is not affected by Munc18-1 constructs. Recovery is plotted as % of input. ***E***, Munc18-1 mutants are not impaired in syntaxin-1 binding. Munc18-1 binding to syntaxin-1 GUVs was determined by analyzing the protein amounts in the sedimented proteo-liposomes by SDS-PAGE. The ratio of surface-exposed syntaxin-1 bound to Munc18-1 wt was set to 100%. Error bars: SD (*n* = 3) of technical replicates. An ordinary one-way ANOVA Dunnett’s test was performed; ****p < 0.0001, ***p < 0.001, n.s., not significant.

These single amino acid substitutions in Munc18-1 did not affect the protein stability as shown by thermal denaturation measurements and size exclusion chromatography (Figs. 1*C*, 2*A*).

Since directly measuring the low affinity binding of VAMP2 to Munc18-1 is difficult because of the requirement of high Munc18-1 concentration, we employed a liposome co-sedimentation assay to test the prediction. This assay capitalizes on the avidity in the presence of multiple template complexes at individual docking sites. To this end, we incubated VAMP2 SUVs (lacking Syt1) with GUVs containing either syntaxin-1 or preassembled Munc18-1*·*syntaxin-1 complexes, respectively. SUVs that are associated with GUVs were isolated after low speed centrifugation (Fig. 2*B*). As expected, in the absence of Munc18-1, VAMP2 SUVs showed inefficient co-sedimentation. The addition of Munc18-1 wt increased co-sedimentation of the VAMP2 SUVs ∼4-fold (also see Parisotto et al., 2012), demonstrating the formation of template complexes (Fig. 2*C*). Munc18-1 Y473D, known to abrogate VAMP2 binding (Meijer et al., 2018), abolished Munc18-1-dependent VAMP2 SUV co-sedimentation (Fig. 2*C*).

In the presence of Munc18-1 Q301D, we did not detect a statistically significant increase in SUV co-sedimentation, indicating that Q301D impairs VAMP2 binding. In contrast, Munc18-1 Q301R increased VAMP2 SUV co-sedimentation 6-fold (Fig. 2*C*). Control experiments showed that the absence or presence of the different Munc18-1 constructs did not the change the amount of sedimented GUVs (Fig. 2*D*). Similar amounts of the Munc18-1 mutants were recovered with the syntaxin-1 GUVs as compared with Munc18-1 wt (Fig. 2*E*). Hence, the Munc18-1 point mutations did not impair the syntaxin-1 binding.

In summary, our data suggest that Munc18-1 Q301D represents a loss of function mutant with reduced VAMP2 affinity and Munc18-1 Q301R embodies a gain of function mutant that exhibits a higher VAMP2 affinity.

### The VAMP2·Munc18-1 interaction is required to promote lipid-mixing in a reconstituted membrane fusion assay

At a functional level, stage-specific inhibitory and stimulatory functions of Munc18-1 can be resolved in reconstituted liposome fusion assays (Shen et al., 2007; Schollmeier et al., 2011; Parisotto et al., 2014; Munch et al., 2016; Sitarska et al., 2017; Meijer et al., 2018). Importantly, the Munc18-1-dependent acceleration of fusion kinetics in such assays strongly depends on the recognition of the cognate v-SNARE, as the direct Munc18-1·VAMP2 interaction stimulates SNARE complex assembly and membrane fusion. In the presence of VAMP8 SUVs stimulatory effects of Munc18-1 have not been observed (Shen et al., 2007; Schollmeier et al., 2011). Consequently, Munc18-1 keeps syntaxin-1 in a closed conformation and the inhibitory function of Munc18-1 becomes prominent (Schollmeier et al., 2011).

**Figure 3.**
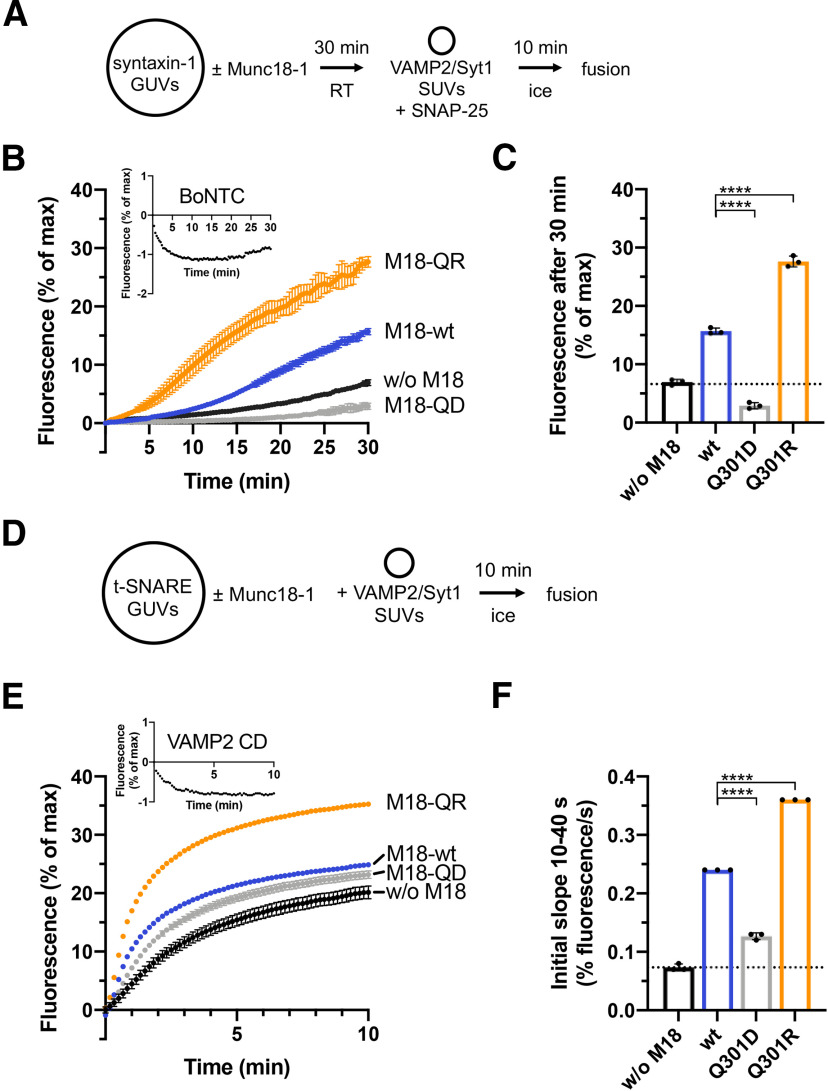
Munc18-1 Q301D and Q301R decrease and increase membrane fusion, respectively. ***A***, Incubation scheme of the syntaxin-1 GUV based lipid mixing assay. ***B***, Munc18-1 Q301R enhances while Q301D inhibits lipid mixing. Lipid mixing kinetics were recorded at 37°C for 30 min as the increase in Atto488 fluorescence. Inset, Representative lipid mixing kinetic showing the negative control containing syntaxin-1 GUVs pretreated with BoNTC, cleaving/inactivating syntaxin-1. Negative controls were subtracted from the fusion reactions. ***C***, Quantification of ***B***. Bar graph represents normalized fluorescence at 30 min (% of max). ***D***, Incubation scheme of the t-SNARE GUV based lipid mixing assay. ***E***, Lipid mixing kinetics were recorded at 37°C for 10 min as the increase in Atto488 fluorescence. Inset, Representative negative control containing an excess of the cytoplasmic domain of VAMP2 (VAMP2 CD), blocking membrane fusion. ***F***, Quantification of ***E***. Bar graph represents the initial slope from 10 to 40 s (% fluorescence/s). Error bars: SD (*n* = 3) of technical replicates. An ordinary one-way ANOVA Dunnett’s test was performed; ****p < 0.0001.

To test the effect of the putative Munc18-1 Q301 gain or loss of function mutants, syntaxin-1 GUVs were preincubated in the absence or presence of Munc18-1 and subsequently VAMP2/Syt1 SUVs together with soluble SNAP-25 were added (Fig. 3*A*). Syt1 assures efficient SUV-GUV docking (Parisotto et al., 2012; Malsam et al., 2012) bypassing potentially rate limiting-docking defects caused by Munc18-1 mutants (e.g., Q301D). The addition of Munc18-1 wt stimulated lipid mixing by a factor of two. Strikingly, Munc18-1 Q301R resulted in a 4-fold stimulation. In contrast, the VAMP2-binding deficient mutant Munc18-1 Q301D lacked the stimulatory effect and fusion was reduced by ∼50% below the level obtained without Munc18-1 addition (Fig. 3*B*,*C*). By starting with GUVs containing preassembled syntaxin-1/SNAP-25 complexes (Fig. 3*D*), the inhibitory function of Munc18-1 is bypassed, because the closed Munc18-1*·*syntaxin-1 complex does not form within the time scale of the preincubation (Meijer et al., 2018). Interestingly, Munc18-1 Q301R still stimulated the lipid mixing kinetics, while Munc18-1 Q301D impaired the stimulatory function (Fig. 3*E*,*F*).

Thus, the interaction of Q301 in Munc18-1 with VAMP2 is critical for Munc18-1 *in vitro* function in SNARE templating. The inhibitory function of Munc18-1 Q301D with syntaxin-1 GUVs confirms that the mutation does not impair the binding of Munc18-1 to closed syntaxin-1.

### Munc13-1 does not bypass the requirement for a Munc18-1·VAMP-2 interaction

**Figure 4.**
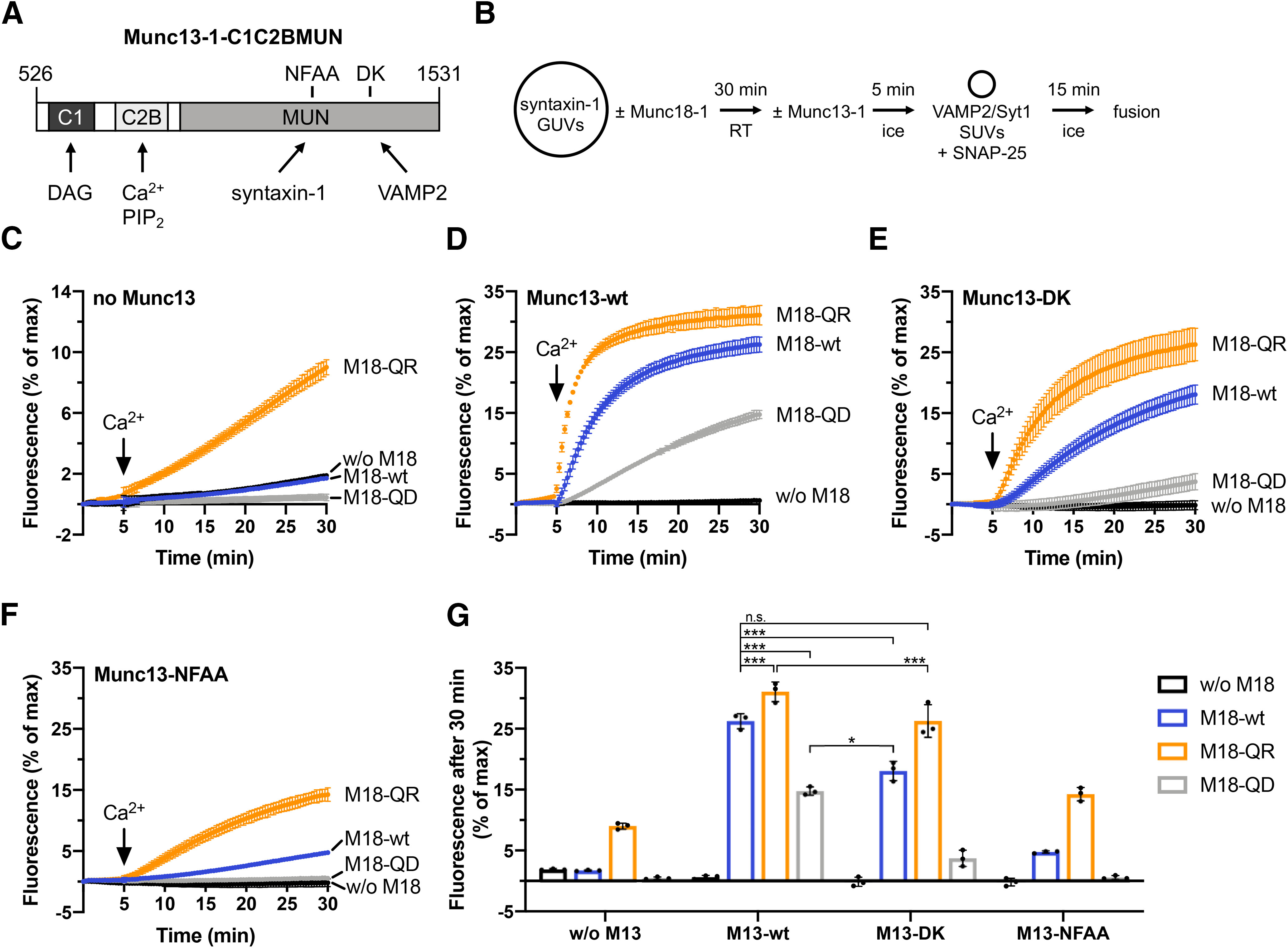
Munc18-1 Q301D and Q301R effects on lipid mixing persist in the presence of Munc13-1-C1C2BMUN. ***A***, Domain scheme of Munc13-1-C1C2BMUN showing protein-protein and protein-lipid interactions and mutations (N1128A/F1131A or D1358K) affecting syntaxin-1 and VAMP2, respectively. ***B***, Incubation scheme of the syntaxin-1 GUV-based lipid mixing assay. GUVs used for measurements with Munc13-1 contained 1 mol% DAG in addition to the standard lipid mixture. Lipid mixing kinetics were recorded at 37°C for 30 min. Measurements were started in the presence of 0.1 mm EGTA. After 5 min, calcium was added to obtain 100 μm free Ca^2+^ in solution. ***C***, Lipid mixing reactions in absence of C1C2BMUN show no calcium response. ***D***, Munc18-1 Q301D (M18-QD) and Q301R (M18-QR) decrease and stimulate Ca^2+^-dependent and C1C2BMUN-dependent lipid mixing, respectively. Please note that the C1C2BMUN-dependent stimulation requires Munc18-1 and Ca^2+^. ***E***, The VAMP2-binding deficient D1358K mutation of Munc13-1 results in a moderate reduction of the overall fusion efficiency. ***F***, Abolished syntaxin-1 interaction of the C1C2BMUN N1128A/F1131A mutant was accompanied by strong inhibition of lipid mixing. ***G***, Quantification of ***C–F***. Bar graphs represent normalized fluorescence after 30 min. Error bars: SD (*n* = 3) of technical replicates. Ordinary one-way ANOVA with Tukey’s test was performed to determine statistical significance and *p* values; *p < 0.05, ***p < 0.001, n.s., not significant.

Recent studies suggest that Munc13-1 assists template complex formation by interacting with VAMP2 and syntaxin-1 (Wang et al., 2019; Shu et al., 2020). This raises the question whether Munc13-1 can bypass the requirement for the Munc18-1·VAMP2 interaction observed in the aforementioned experiments. To address this, we tested the effects of a Munc13-1 construct comprising the lipid-interacting C1 and C2B domains and the MUN domain (C1C2BMUN) or mutants thereof in the lipid mixing assay (Fig. 4*A*). For efficient activation of Munc13-1, GUVs contained 1 mol% DAG in addition to the standard lipid composition and 100 μm free calcium were added during the reaction (Fig. 4*B*). Addition of C1C2BMUN wt led to strong stimulation of lipid mixing in response to calcium addition (compare Fig. 4*C*,*D*,*G*). This stimulation was strictly Munc18-1 dependent. Remarkably, Munc18-1 Q301R still stimulated lipid-mixing while Munc18-1 Q301D retained its inhibitory function relative to Munc18-1 wt (Fig. 4*D*). Similar effects of the Munc18-1 constructs on lipid mixing were also observed in measurements using the syntaxin-1 binding mutant C1C2BMUN N1128A/F1131A (NFAA) or the VAMP2 binding mutant C1C2BMUN D1358K (DK; Fig. 4*E–G*). However, the overall fusion efficiency was moderately or strongly reduced by the DK or the NFAA mutant, respectively. The individual C1C2BMUN DK and Munc18-1 Q301D mutants impaired membrane fusion overall to a similar degree with the Munc18-1 Q301D effect being slightly more pronounced (compare Fig. 4*D*, gray line, and *E*, blue line, *G*). Furthermore, stimulation by Munc18-1 Q301R rescued the inhibitory effect of C1C2BMUN DK to wt levels (compare Fig. 4*D*, blue curve, and *E*, orange curve, *G*), which was not the case for the syntaxin-1 binding deficient Munc13-1 NFAA mutant (Fig. 4*F*). Overall, these findings suggest synergistic effects for VAMP2- and Munc13-1 binding in generating the template complex.

### Munc18-1 Q301D inhibits exocytosis in mouse hippocampal neurons

**Figure 5.**
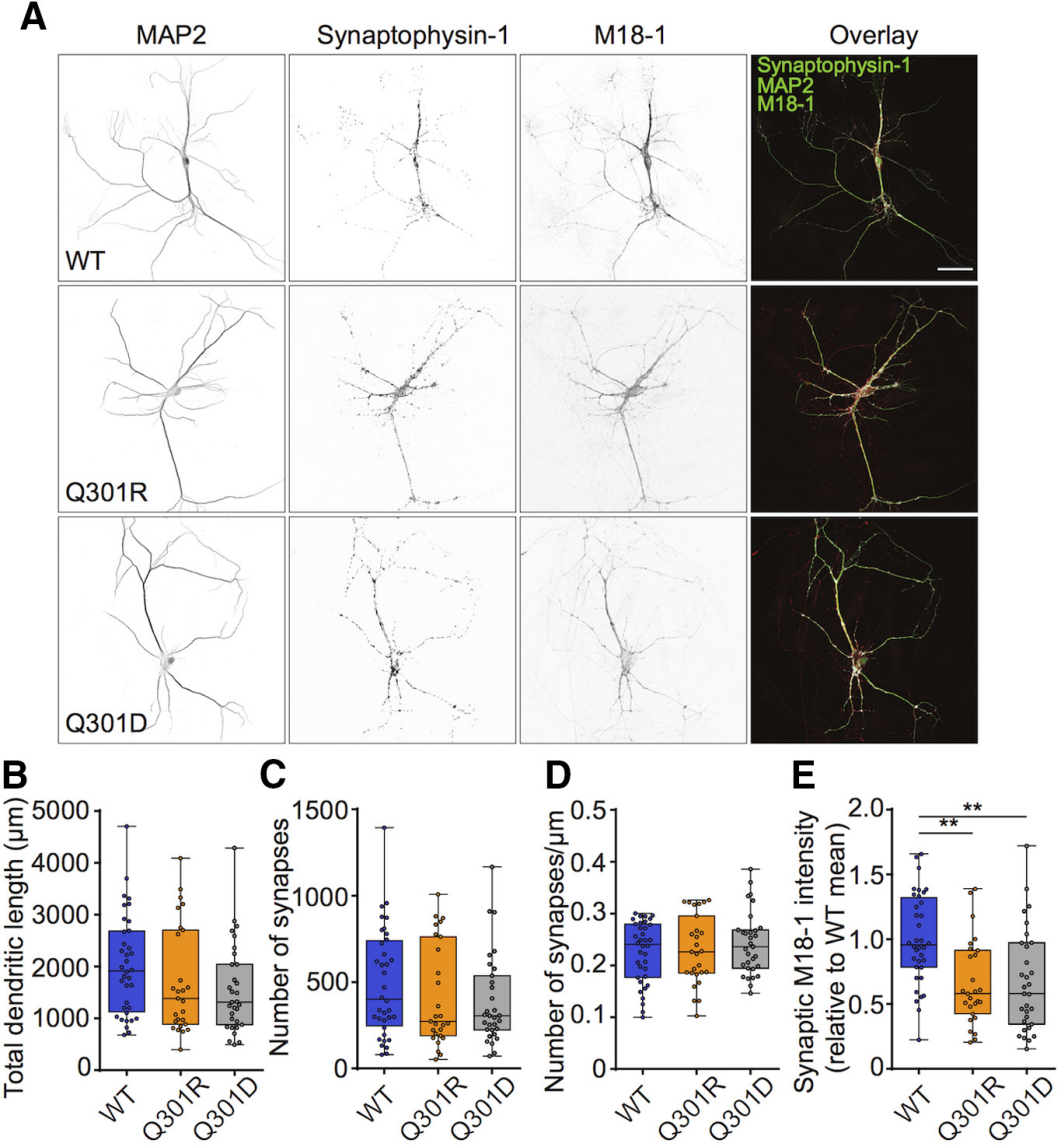
Effects of the Munc18-1 variants on neuronal morphology and synaptic Munc18-1 levels. ***A***, Example confocal images of the staining of autaptic neurons from the three conditions, showing the intensity of MAP2, Synaptophysin-1, and M18-1 stainings separately (inverted greyscale images, first three columns) and overlaid (fourth column). Green, MAP2; cyan, Synaptophysin-1; red, M18-1. Scale bar = 50 μm. ***B***, Median [Q1–Q3] total dendritic length of neurons (M18WT: 1915 [1126–2686] μm, *n* = 36; M18Q301R: 1384 [886.5–2704] μm, *n* = 27; M18Q301D: 1310 [878.3–2042] μm, *n* = 31; Kruskal–Wallis test, *p* = 0.0922). ***C***, Median [Q1–Q3] number of synapses (M18WT: 401 [246.5–740.8], *n* = 36; M18Q301R: 273 [189–763], *n* = 27; M18Q301D: 305 [223–537], *n* = 31; Kruskal–Wallis test, *p* = 0.3762). ***D***, Median [Q1–Q3] number of synapses per dendritic length (M18WT: 0.24 [0.18–0.28] μm^–1^, *n* = 36; M18Q301R: 0.23 [0.19–0.30] μm^–1^, *n* = 27; M18Q301D: 0.24 [0.19–0.27] μm^–1^, *n* = 31; Kruskal–Wallis test, *p* = 0.7669). ***E***, The relative synaptic M18-1 intensity was calculated by dividing the average M18-1 intensity in synaptophysin puncta per neuron by the mean value of the M18WT condition of the corresponding culture. Median [Q1–Q3] relative synaptic M18-1 intensity (M18WT: 0.96 [0.78–1.32], *n* = 36; M18Q301R: 0.58 [0.42–0.91], *n* = 27; M18Q301D: 0.58 [0.35–0.97], *n* = 31; Kruskal–Wallis test, ***p = 0.0003, Dunn’s multiple comparison test, M18WT vs M18Q301R: **p = 0.0019, M18WT vs M18Q301D: **p = 0.0014, M18Q301R vs M18Q301D: *p* > 0.9999).

Finally, to test the physiological relevance, we analyzed to what extent the Munc18-1Q301 mutants support synaptic transmission in living neurons. *Munc18-1* null neurons expressing Cre-EGFP plus either Munc18-1 wt, Munc18-1 Q301R, or Munc18-1 Q301D using lentiviral vectors were all viable, unlike uninfected *munc18-1* null neurons (Santos et al., 2017), and all showed normal *in vitro* morphology, total dendritic length, and synapse density (Fig. 5*A–D*). Munc18-1 Q301R and Munc18-1 Q301D were expressed at lower levels in the synapses than Munc18-1 wt (Fig. 5*E*). Hence, both mutants produce functional proteins, which are, despite a lower synaptic expression level, sufficient to support neuronal viability, and normal synaptogenesis. The lower expression levels are unlikely to affect synaptic transmission per se, as previous studies showed that a 50% reduction does not affect basic synaptic transmission in the same model system (Toonen et al., 2006). Moreover, the fact that the two mutants, which have opposite effects in cell free assays (see above), are expressed at similar levels in hippocampal synapses, allows a stringent comparison *in situ*.

**Figure 6.**
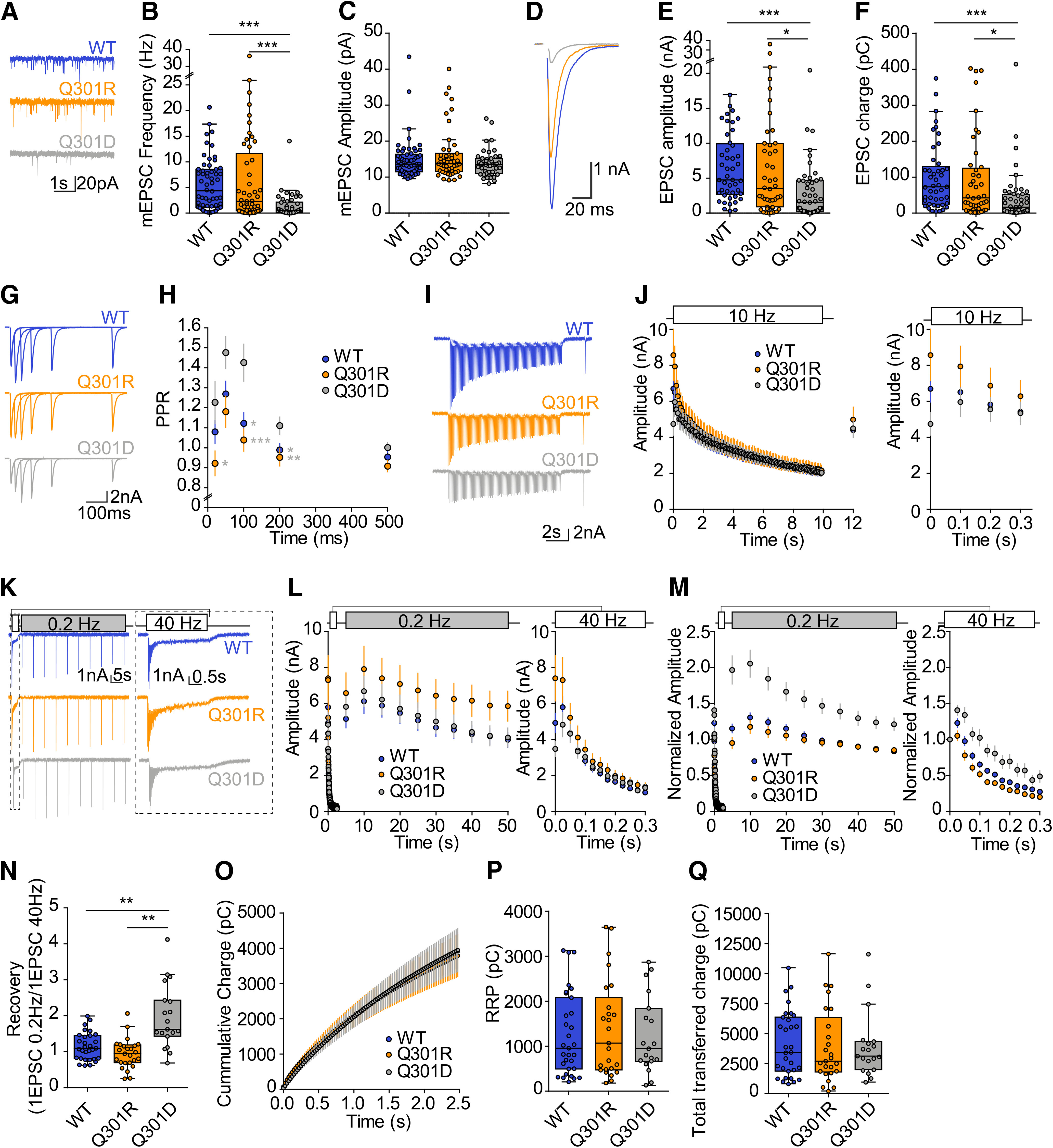
Munc18-1 Q301D drastically impairs synaptic transmission while Q301R supports normal transmission. ***A***, Typical examples of passive recordings showing mEPSCs. ***B***, mEPSC frequency (M18WT: 4.40 Hz, *n* = 58; M18Q301R: 2.29 Hz, *n* = 49; M18Q301D: 0.517 Hz, *n* = 46; *p* < 0.0001, M18WT vs M18Q301R: *p *>* *0.05, M18WT vs M18Q301D: *p* < 0.001, M18Q301R vs M18Q301D: *p *<* *0.001). ***C***, mEPSC amplitude (M18WT: 13.61 pA, *n* = 58; M18Q301R: 13.74 pA, *n* = 47; M18Q301D: 13.50 pA, *n* = 43; *p *=* *0.50, M18WT vs M18Q301R: *p *>* *0.05, M18WT vs M18Q301D: *p* > 0.05, M18Q301R vs M18Q301D: *p > *0.05). ***D***, Example traces of evoked EPSCs. ***E***, Evoked EPSC amplitude (M18WT: 4.78 nA, *n* = 45; M18Q301R: 3.57 nA, *n* = 44; M18Q301D: 1.60 nA, *n* = 48; *p *=* *0.0001, M18WT vs M18Q301R: *p *>* *0.05, M18WT vs M18Q301D: *p* < 0.001, M18Q301R vs M18Q301D: *p *<* *0.05). ***F***, Evoked EPSC charge (M18WT: 71.91 pC, *n* = 45; M18Q301R: 40.92 pC, *n* = 44; M18Q301D: 15.38 pC, *n* = 48; *p *<* *0.0001, M18WT vs M18Q301R: *p *>* *0.05, M18WT vs M18Q301D: *p* < 0.001, M18Q301R vs M18Q301D: *p *<* *0.05)**. *G***, Typical examples of paired pulse traces. Traces from different intervals are superimposed (20, 50, 100, 200, and 500 ms). ***H***, Quantification of the paired pulse ratio for different intervals (M18WT: *n* = 37; M18Q301R: *n* = 29; M18Q301D: *n* = 23). ***I***, Typical examples of 10-Hz stimulation train. ***J***, Quantification of EPSC charge during 10-Hz train stimulation. Followed by a single stimulation to monitor post-stimulation response (M18WT: *n* = 34; M18Q301R: *n* = 29; M18Q301D: *n* = 21). Right, Insert of the first four pulses. ***K***, Typical examples of 40-Hz stimulation train. Right, Insert of the 40-Hz train stimulation responses. ***L***, Quantification of EPSC amplitude during 40-Hz train stimulation. Followed by a 0.2-Hz train stimulation to monitor post-stimulation recovery (M18WT: *n* = 31; M18Q301R: *n* = 25; or M18Q301D: *n* = 19). Right, Insert of the first 300 ms. ***M***, Normalized EPSC amplitude quantification during 40-Hz train stimulation and 0.2 Hz. The 0.2-Hz recovery train EPSC amplitude is normalized to the first EPSC of the 40-Hz train stimulation (M18WT: *n* = 31; M18Q301R: *n* = 25; or M18Q301D: *n* = 19). Right, Insert of the first 300 ms. ***N***, Recovery of EPSC amplitude. First pulse of the 0.2-Hz train (M18WT: 1.11, M18Q301R: 0.95, M18Q301D: 1.631, *p *<* *0.0001; M18WT vs M18Q301R: *p *>* *0.05, M18WT vs M18Q301D: *p* < 0.01, M18Q301R vs M18Q301D: *p *<* *0.001). ***O***, Cumulative EPSC charge during 40-Hz train stimulation. ***P***, Quantification of the RRP based on back-extrapolation to the *y*-axis of a linear fit through the last 20 points of the cumulative EPSC charge (M18WT: 951.4 pC, M18Q301R: 1066 pC, M18Q301D: 942.1 pC, *p *=* *0.97). ***Q***, Quantification of total cumulative charge transferred during 40-Hz train stimulation (M18WT: 3442 pC, M18Q301R: 2711 pC, M18Q301D: 3133 pC, *p *=* *0.87). Medians are reported. Statistically tested using the Kruskal–Wallis test and Dunn’s multiple comparison test. *p <* *0.05, **p <* *0.01, ***p <* *0.001.

Using whole-cell patch-clamp electrophysiology on single hippocampal neurons grown on glia islands (autapses), synaptic transmission and short-term plasticity were assessed in all three experimental groups (Q301R, Q301D and wt). Neurons expressing Munc18-1 Q301R synapses showed a normal miniature EPSC (mEPSC) frequency, while Q301D showed a significantly lower frequency (Fig. 6*A*,*B*). The mEPSC amplitude was similar for all groups (Fig. 6*C*). Evoked EPSC amplitudes and charge were again similar to wt for the Q301R-expressing synapses, while Q301D-expressing synapses showed a 3-fold reduction (Fig. 6*D–F*). Paired pulse plasticity was evaluated with varying inter-stimulus intervals and showed that neurons expressing Munc18-1 Q301D had significantly higher paired pulse ratio compared with neurons expressing Munc18-1 Q301R at intervals of 20, 100, and 200 ms and compared with neurons expressing Munc18-1 wt at intervals of 10 and 200 ms (Fig. 6*G*,*H*). To evaluate the response to high frequency stimulation, we applied 10-Hz (Fig. 6*I*,*J*) and 40-Hz (Fig. 6*K*,*M*) train stimulation. While the differences in initial evoked responses were confirmed (Fig. 6*J–L*, right panels), no difference in run down kinetics was observed between the three groups. To assess synaptic recovery after exhaustive stimulation, the 40-Hz train was followed by 0.2-Hz stimulation. Neurons expressing Munc18-1 Q301D showed a relatively faster and stronger recovery, almost twice the first evoked pulse of the 40-Hz train (Fig. 6*M*,*N*). The estimated RRP size, as assessed by back-extrapolation of the steady state recruitment rate using the cumulative charge during the 40-Hz train (Fig. 6*O*), as well as the total transferred charge did not differ between the three conditions (Fig. 6*O–Q*).

In conclusion, neurons expressing Munc18-1 Q301R support synaptic transmission and plasticity similar to Munc18-1 wt, while neurons expressing Munc18-1 Q301D have a severely reduced release probability, as indicated by the 3-fold reduced evoked EPSCs and increased paired pulse ratio. However, recovery after synaptic depletion was relatively fast in Munc18-1 Q301D-expressing neurons.

## Discussion

In this study, we used a combination of comparative structure modeling (Fig. 1*A*) and site-specific crosslinking and mutagenesis (Fig. 1*B*,*D*) to precisely map the binding site for VAMP2 on the surface of Munc18-1, cell free assays to test the consequence of mutations in fusion reactions and synapse physiology to test *in vivo* relevance. Importantly, the analyses were performed with full-length syntaxin-1 and VAMP2 reconstituted into liposomes and their binding partner Munc18-1 bridging the membranes. The crosslink pattern, the GUV-SUV binding (Fig. 2) and membrane fusion studies (Fig. 3) demonstrate that the binary Munc18-1*·*syntaxin-1 complex provides a specific binding site for VAMP2 and that the interface is localized to helix 11 and 12 of Munc18-1.

The crosslinks also reveal that the loop connecting helix 11 and 12 of Munc18-1 needs to be in its unfurled conformation for the association of VAMP2 (also see Sitarska et al., 2017). Munc18-1·VAMP2 crosslinks at L327, M330 and especially L331 and L341 are not possible in the furled loop conformation. The findings suggest that the binding interface covers VAMP2 residues between layers −4 to +4. The observation that A297Bpa and M355Bpa impair Munc18-1 function, in the absence of crosslinking, extends the interface to the +5 area. Interestingly, A297 and M355 are in close proximity to Y473. The Y473D mutant causes a strong loss of function phenotype in lipid mixing assays and strongly impairs synaptic transmission in living neurons (Meijer et al., 2018). Our model also suggests that residue L247 contributes to the binding of F77 (layer +6) of VAMP2, which was previously shown experimentally (Jiao et al., 2018; Wang et al., 2019). Furthermore, the inhibitory and stimulatory phenotypes observed with Munc18-1 Q301D/R mutants are likely the direct consequence of altered VAMP2 D68 interactions, further suggesting that the molecular model correctly displays the binding interface.

Our results are consistent with recent biophysical single molecule studies, revealing how Munc18-1 controls the order of events and the molecular forces occurring during neuronal SNARE complex zippering (Jiao et al., 2018). Thus, the template complex likely provides the starting point for subsequent SNAP-25 binding and further SNARE complex assembly. Apparently, Vps33 and Munc18-1 employ similar structural principles to interact with Nyv1 and VAMP2, respectively, indicating that SM proteins use a conserved mechanism to stimulate N-terminal Qa/R-SNARE assembly (also see Jiao et al., 2018; Torng et al., 2020).

Interestingly, we also observed a stimulatory role for Munc18-1 when preassembled syntaxin-1*·*SNAP-25-SNARE complexes were reconstituted into GUVs. This indicates that Munc18-1 bound to the t-SNARE complex (Dawidowski and Cafiso, 2016) interacts with VAMP2 and still stimulates SNARE complex assembly (Shen et al., 2007; Shi et al., 2011; Jakhanwal et al., 2017). However, a Munc18-1*·*syntaxin-1*·S*NAP-25 complex may not represent a major intermediate along the physiological pathway as it has been shown that Munc18-1 dissociates the syntaxin-1*·*SNAP-25 complex forming the very stable closed Munc18-1*·*syntaxin-1 complex (Ma et al., 2013). Importantly, several additional regulatory components such as Munc13-1, complexins, Syt1, α-SNAP and NSF likely orchestrate SNARE complex assembly preventing the formation of unproductive intermediates and promoting forward directionality (He et al., 2017; Lai et al., 2017; Jiao et al., 2018).

Both the reconstituted membrane fusion assay (Figs. 3, 4) and the electrophysiological studies in living neurons (Figs. 5, 6) reveal a critical role for Munc18-1 Q301 in SNARE complex assembly further confirming the physiological relevance of the structural model. The Q301D mutant impairs lipid mixing, consistent with the reduction of mEPSC and evoked release. However, the gain of function phenotype of Q301R was observed in the GUV-SUV fusion assay, but not in synapse physiology. Thus, it appears that in the reconstituted assay Q301R stimulates a step in template complex formation, which may not be rate-limiting in the cellular context. Munc13-1 addition to the liposome fusion assay did not resolve this discrepancy because Q301R still shows a significant stimulation on lipid mixing kinetics in the presence of Munc13-1. Furthermore, other gain of function mutations in Munc18-1 such as P335A, which directly causes helix 12 extension, do show a prominent phenotype in secretory vesicle fusion in chromaffin cells (Munch et al., 2016). However, introduction of other single amino acid mutations in Munc18-1 studied in the same model system as employed here (single mouse hippocampal neurons), showed a robust increase in synaptic transmission (Lammertse et al., 2020) excluding a ceiling effect of the assay. Taken together, these considerations suggest that *in situ* conditions in the synapse are different from *in vitro* reconstituted conditions and prevent a gain-of-function phenotype in the former, but not in the latter case.

Overall, employing crosslinking technology, which detects weak and transient interactions during the priming of the fusion machinery, embedded in its natural membrane environment, we resolved the Munc18-1·VAMP2 interface at the structural level. We showed that, on unfurling of the Munc18-1 loop, helix 11 and 12 in domain 3a bind to the central region of the VAMP2 SNARE motif, and validated its functional relevance in reconstituted assays and in living neurons. Together, these data establish the molecular determinants of how Munc18-1 executes SNARE-templating.

## Synthesis

Reviewing Editor: Quentin Pittman, University of Calgary Decisions are customarily a result of the Reviewing Editor and the peer reviewers coming together and discussing their recommendations until a consensus is reached. When revisions are invited, a fact-based synthesis statement explaining their decision and outlining what is needed to prepare a revision will be listed below. The following reviewer(s) agreed to reveal their identity: Colin Johnson. Note: If this manuscript was transferred from JNeurosci and a decision was made to accept the manuscript without peer review, a brief statement to this effect will instead be what is listed below.

This well-written manuscript describes a set of very interesting findings related to SNARE assembly and neurotransmitter release. Increasing evidence suggests that Munc18-1 can function as a template to line up VAMP2 and syntaxin-1 for efficient assembly. The structural details have, however, proven difficult to figure out, particularly as regards the putative binding of VAMP2 to Munc18-1. Here, the authors use a clever new strategy involving cross-linking via non-natural amino acids incorporated into Munc18-1 to provide strong support for their homology-based structural model of the Munc18-1:VAMP2 ‘template complex’. A particular strength of this analysis is that it was performed using liposome-anchored syntaxin-1 and VAMP2 to mimic the ‘trans-SNARE’ configuration.

The remainder of the manuscript concerns mutations at Munc18-1 residue 301 that either enhance (Q301R) or compromise (Q301D) VAMP2 binding. The mutant Munc18-1 proteins are deployed in a diverse set of in vitro liposome “fusion” (actually lipid mixing) assays. The most important implication, in my view, is that Munc18-1:VAMP2 templating is critical in all contexts, with or without Munc13-1, and even if the starting point for the Qa-SNARE is not a complex with Munc18-1 (which is probably the physiologically relevant starting point) but also if it is a complex with SNAP-25. The authors round out their study by investigating the impact of Q301R and Q301D in living neurons. In good agreement with their findings in vitro, Q301D causes a reduction in evoked neurotransmitter release. The only unresolved puzzle is why Q301R doesn’t exhibit a gain-of-function phenotype. This is likely telling us something interesting (but beyond the scope of the current manuscript). Overall, the manuscript makes a strong contribution to the literature.

Overall the reviewers and I found that the manuscript is well-written and the statistical analysis the was performed appears reasonable. You have addressed the questions raised in a previous round of review to our satisfaction. We were impressed with the quality of the presentation.

There were 2 minor suggestions for revision as follows:

1. That the authors should incorporate parts of their answer to why % fusion in Figure 4C differs by such a large amount compared to Figure 3B into the main text, perhaps the methods section ?

2. As a suggestion, the authors may want to incorporate Additional Figure 2 in the rebuttal letter as inset figure panels in the manuscript.

Once I have received a revised version of the manuscript I will make a decision without further review.
